# Association of cholinesterase activities and POD in older adult abdominal surgical patients

**DOI:** 10.1186/s12871-022-01826-y

**Published:** 2022-09-16

**Authors:** Zdravka Bosancic, Claudia D. Spies, Anika Müller, Georg Winterer, Sophie K. Piper, Maria Heinrich, on behalf of the BioCog Consortium

**Affiliations:** 1 Charité – Universitätsmedizin Berlin, Corporate Member of Freie Universität Berlin and Humboldt Universität zu Berlin and Berlin Institute of Health, Department of Anesthesiology and Operative Intensive Care Medicine (CCM, CVK), Augustenburger Platz 1, 13353 Berlin, Germany; 2 Pharmaimage Biomarker Solutions GmbH, Berlin, Germany; 3 Charité – Universitätsmedizin Berlin, Corporate Member of Freie Universität Berlin, Humboldt-Universität zu Berlin, and Berlin Institute of Health, Experimental and Clinical Research Center (ECRC), Berlin, Germany; 4 Charité – Universitätsmedizin Berlin, Corporate Member of Freie Universität Berlin, Humboldt-Universität zu Berlin, and Berlin Institute of Health, Institute of Biometry and Clinical Epidemiology, Charitéplatz 1, 10117 Berlin, Germany; 5 Berlin Institute of Health at Charité – Universitätsmedizin Berlin, Charitéplatz 1, 10117 Berlin, Germany; 6 Charité – Universitätsmedizin Berlin, Corporate Member of Freie Universität Berlin, Humboldt-Universität zu Berlin, and Berlin Institute of Health, Institute of Medical Informatics, Charitéplatz 1, 10117 Berlin, Germany; 7 Berlin Institute of Health at Charité – Universitätsmedizin Berlin, BIH Biomedical Innovation Academy, BIH Charité (Junior) (Digital) Clinician Scientist Program, Charitéplatz 1, 10117 Berlin, Germany

**Keywords:** POD, Postoperative, Older adults, Cholinesterase activity

## Abstract

**Background:**

Postoperative delirium (POD) is a frequent complication after surgery. Older adult patients undergoing abdominal surgery are at higher risk for developing POD. Studies on the association of cholinesterase activities and POD are rare, but leading hypotheses implicate that the cholinergic pathway might play an important role in neuroinflammation and development of POD. The objective of this study was to figure out if there is an association between the development of POD and acetyl- and butyrylcholinesterase (AChE and BuChE) activities in older adult patients undergoing abdominal surgery.

**Methods:**

The investigation was performed with a subpopulation of BioCog study patients. The BioCog project (http://www.biocog.eu) is a prospective multicenter observational study in older adult surgical patients. Patients ≥ 65 years undergoing elective surgery of at least 60 minutes who scored more than 23 points in the Mini-Mental-State-Examination were included. POD was assessed twice a day on seven consecutive days after the surgery, using the test instruments Nursing Delirium Screening Scale (Nu-Desc) and Confusion Assessment Method (CAM and CAM-ICU) and a patient chart review. Pre- and postoperative blood cholinesterase activities were measured with a photometric rapid-point-of-care-testing. The association between cholinesterase activities and POD was analyzed in a subpopulation of abdominal surgical patients using multivariable logistic regression analysis adjusting for confounders.

**Results:**

One hundred twenty-seven patients were included for analysis (mean age 73 years, 59% female). Fifty-two patients (41%) fulfilled the criteria of POD. These patients were significantly older, had a longer time of surgery and anesthesia and achieved higher comorbidity scores compared to patients without POD. After adjusting for age, duration of surgery and charlson comorbity index, we found an association between pre- and postoperative AChE activity (U/gHb) and the development of POD (Odds ratio (OR), [95% confidence interval (CI)], preoperative 0.95 [0.89–1.00], postoperative 0.94 [0.89–1.00]).

**Conclusions:**

We found an association between POD and AChE activity and provided new information considering patients with abdominal surgery. Future analyses should examine course dynamics of postoperative cholinesterase activities in order to clarify interactions between the cholinergic system and pathophysiological mechanisms leading to POD.

**Trial registration:**

ClinicalTrials.gov: NCT02265263.

**Supplementary Information:**

The online version contains supplementary material available at 10.1186/s12871-022-01826-y.

## Background

Postoperative delirium (POD) describes an acute, fluctuating neurocognitive dysfunction occurring after surgery. Patients suffer from altered consciousness and orientation as well as experience a decline in other neurocognitive functions [1]. It is associated with higher mortality rates, long-term cognitive impairment and other long-term consequences [2–4]. Risk factors can be classified in predisposing (e.g. age) and precipitating factors (e.g. site of surgery) [5]. Patients with abdominal surgery are at higher risk of developing POD [6]. Pathophysiological pathways are not conclusively clarified.

Inflammatory interactions between the parasympathetic system and the abdominal cavitiy might modulate neuroinflammatory processes [7, 8]. Dysbalanced neuroinflammatory reactions might play an important role in the pathophysiology of POD and lead to long term consequences such as neurodegeneration [9]. The cholinergic approach offers an explanation for the dysbalance leading to excessive neuroinflammation [10].

Acetylcholine (ACh) seems to reduce inflammatory processes in the brain as well as systemic responses [8, 11, 12]. A dysregulation in cholinergic neurotransmitter systems might lead to a decrease of inhibitory effects and thus contribute to increased neuroinflammatory reactions [13]. Genetic variants of cholinergic genes seem to be associated with the incidence of POD [14]. Measuring the amount of ACh in the synaptic cleft or in the blood plasma is complex and often impossible [15, 16]. However, activity of cholinesterases in the peripheral blood, acetyl- and butyrylcholinesterase (AChE and BuChE) activities, has been measured in previous studies to assess fluctuations and courses of cholinergic metabolism [15]. The enzymes AChE and BuChE both catalyze the hydrolysis of choline esters. AChE is mainly located in brain tissue, hematopoetic cells and muscle cells and has a much higher turnover rate compared to BuChE [17]. BuChE can be found in most tissues and body fluids, has a lower catalytic efficiency but contributes to ACh homeostastis and immune modulation [18].

The aim of this investigation was to clarify if acetyl- and butyrylcholinesterase activities are associated with POD in older adult patients undergoing abdominal surgery.

## Methods

### Study design and population

This investigation is part of the BioCog project (www.biocog.eu), a prospective multicenter observational study conducted between October 2014 and June 2019 in Berlin, Germany and Utrecht, Netherlands. Male and female patients aged ≥ 65 years undergoing elective surgery (> 60 min), achieving a Mini Mental State Examination (MMSE) score of at least 23 points, were included. For complete inclusion and exclusion criteria see previous publications [19].

For this subanalysis, we studied patients who underwent abdominal surgery and had complete pre- and postoperative cholinesterase measurements. The BioCog study was approved by the local ethics committees (ref. EA2/092/14 and 14–469) and conducted compliant with the declaration of Helsinki (ClinicalTrials.gov.: NCT02265263, 15/10/2014) as well as all local data privacy regulations. Written informed consent was obtained from all participating patients.

### Baseline measurements

The following baseline measurements were assessed to describe study population: age, sex, MMSE, Body-Mass-Index (BMI), physical status according to the American Society of Anesthesiologists (ASA PS), Charlson Comorbidity Index (CCI) [20], preoperative neurocognitive disorder (for details see [19]), impaired activities of daily living according to Barthel (ADL) [21] as well as Lawton and Brody (IADL) [22], malnutrition according to Mini-Nutritional Assessment (MNA) [23], preoperative frailty status (for details see [19]), smoking status, alcohol consumption according to AUDIT (Alcohol Use Disorders Identification Test) [24], preoperative depression according to geriatric depression scale (GDS) [25], malignant tumor surgery, preoperative and intraoperative anticholinergic load, calculated using the Anticholinergic Drug Scale (ADS) [26], premedication with midazolam, type of anesthesia (general vs. regional vs. combined), intraoperative clonidine or norepinephrine administration, duration of surgery and duration of anesthesia, stay on intensive care unit (ICU) and pre- and postoperative CRP level and leukocyte counts.

### Cholinesterase measurements

Blood samples were taken on the morning of the surgery and on the first postoperative day exclusively via venipuncture. Due to external conditions (e.g. working times of the laboratory) 19 of the 127 patients (15%) had their first postoperative blood sample taken on the third postoperative day.

A photometric rapid-point-of-care testing was used to measure pre- and postoperative cholinesterase activities (erythrocyte AChE and free plasma BuChe activities) (ChE check mobile®, Securetec Detektions-Systeme AG, Neubiberg, Germany) [27]. The testing was performed within 1 h after collecting the blood sample.

### Postoperative delirium

POD was defined according to the criteria of the Diagnostic and Statistical Manual of Mental disorders 5th Edition [1]. The screening was performed twice a day on seven consecutive days after the surgery independently of the routine hospital procedures. The assessment was conducted by a research team (study physicians, doctoral students, study nurses), which were trained and supervised by psychiatrists and delirium experts.

Patients were considered delirious when achieving ≥ 2 cumulative points on the Nursing Delirium Screening Scale (NuDesc) AND/OR a positive Confusion Assessment Method (CAM) score AND/OR a positive CAM for the Intensive Care Unit (CAM-ICU) score AND/OR descriptions of delirium on patient chart review (e.g. confused, agitated, drowsy, disorientated, delirious, or received antipsychotic therapy for delirium).

POD was classified into a hypoactive, hyperactive or mixed form in accordance with preliminary work on the study of delirium subtypes [28]. For hypoactive POD result of the Richmond Agitation-Sedation Scale (RASS) was between 0 and − 3 and for hyperactive between + 1 and + 4. A mixed form of POD was defined with both positive and negative RASS values [28].

### Statistical analysis

**Fig. 1:**
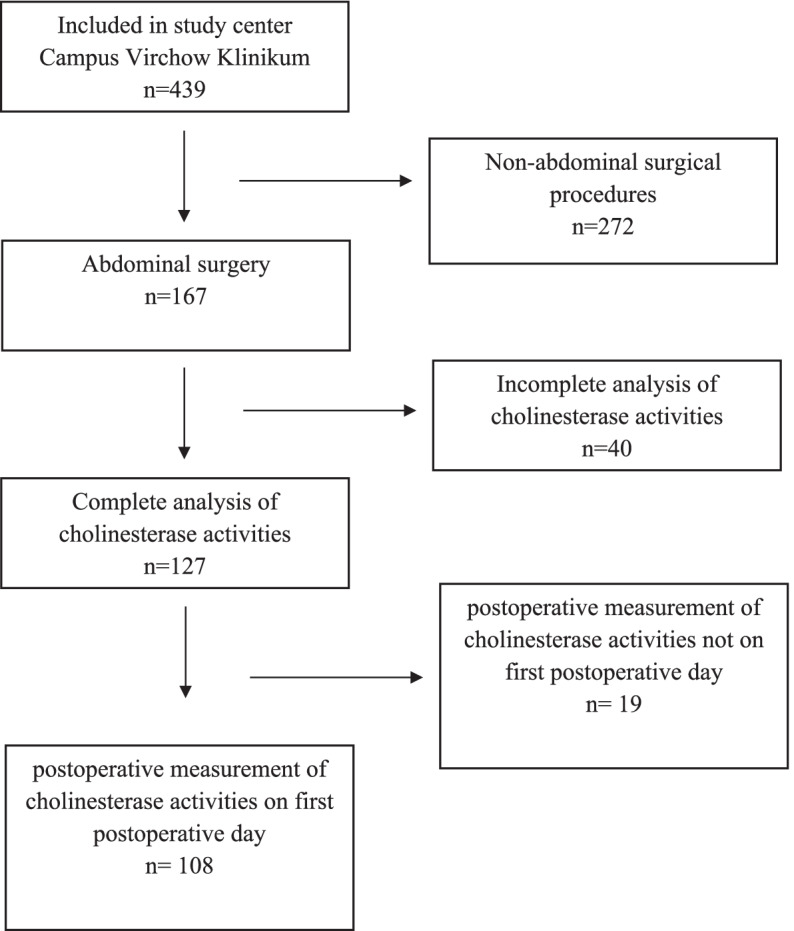
Flow chart

Baseline characteristics were expressed as median and 25th percentile and 75th percentile, mean ± standard deviation (SD) or frequencies with percentages. Differences between the groups were tested using the Mann-Whitney U test, Kruskal Wallis Test, the t-test or chi-squared test, depending on distribution and scale. For improved visualization, the values for BuChe-activity (U/l) were scaled with factor 0.01 (100 U/l). The associations between POD and cholinesterase activities were analyzed via multivariable logistic regression after adjusting for possible confounding variables. Confounding variables were selected a priori: age, duration of surgery, CCI. The t-test was used to examine whether the mean values of the postoperative cholinesterase activity measurements on postoperative day 1 (*n* = 108) differed significantly from the mean values of the measurements on postoperative day 3 (*n* = 19) (see Fig. 1). Logistic regression analysis was repeated with the smaller group of 108 patients, having their postoperative blood samples taken exclusively on the first postoperative day (*n* = 108). Significance level alpha was set to 0.05. No adjustments were made for multiple testing. All *p*-values constitute exploratory data analysis. All analyses were performed with SPSS Statistics, version 24 (©1989, 2016 by SPSS Inc., Chicago, Illinois, USA).

## Results

A total of 127 patients undergoing abdominal surgery with pre- and postoperative AChE and BuChE activity measurements were included in the analysis (see Fig. 1). Fifty-two patients (41%) suffered from POD. These patients were statistically significantly older, achieved higher ASA-PS and CCI scores, showed more often frailty phenotype, had a significantly longer duration of surgery and anesthesia, underwent more often malignant tumor surgery, needed more often ICU treatment and showed higher pre- and postoperative CRP levels (see Table 1). Regarding sex, MMSE, BMI, preoperative anticholinergic load, smoking status, alcohol consumption, preoperative cognitive disorder, activities of daily living (ADL and IADL), malnutrition, GDS, leukocyte counts, type of anesthesia or drug administration there were no significant differences between patients with and without POD. Thirty-five patients of delirious patients (71%) showed a hypoactive, 5 (10%) a hyperactive and 5 (10%) a mixed form of POD. For 5 patients there is no information on delirium subtype.

**Table 1:** Patient characteristics (*n* = 127)

Characteristic	POD *n* = 52 (41%)	No POD *n* = 75 (59%)	*P*-value
Age (years)	73 (71;77)	72 (69;75)	0.023^a^
female sex	30 (58%)	45 (60%)	0.795^b^
MMSE (points)	29 (27;30)	29 (28;30)	0.316^a^
BMI (kg/m^2^)	26 (23; 29)	26 (23;29)	0.533^a^
ASA-PS			
I	1 (1.9%)	2 (2.7%)	0.042^b^
II	22 (42.3%)	48 (64%)	
III	29 (55.8%)	25 (33.3%)	
CCI (points)	3 (2;3)	2 (1;3)	0.016^a^
Frailty status^c^			
Robust	9 (17.6%)	38 (51.4%)	
Pre-frail	28 (54.9%)	31 (41.9%)	< 0.001^b^
Frail	14 (27.5%)	5 (6.8%)	
Preoperative neurocognitive disorder^c^			
Mild	12 (27.9%)	7 (11.5%)	0.093^b^
Major	2 (4.9%)	5 (8.2%)	
ADL impaired^c^	13 (25.5%)	11 (14.9%)	0.138^b^
IADL impaired^c^	7 (14.6%)	5 (7.7%)	0.240^b^
Preoperative anticholinergic load	10 (19.2%)	13 (17.6%)	0.812^b^
MNA^c^			
Risk for malnutrition	20 (39.2%)	20 (27.0%)	0.310^b^
Malnutrition	6 (11.8%)	8 (10.8%)	
Smokers^c^	7 (14.9%)	3 (4.5%)	0.056^b^
Alcohol consumption^c^			
Normal	42 (89.4%)	61 (93.8%)	0.389^b^
Hazardous	5 (10.6%)	4 (6.2%)	
GDS (points)	1.2 (0;3.2)	1 (0;3.1)	0.615^a^
Malignant tumor surgery	46 (88.5%)	52 (69.3%)	0.012^b^
Duration of surgery (min)	312 (190;393)	196 (120;301)	0.001^a^
Premedication with midazolam	7 (13.5%)	18 (24.0%)	0.142^b^
Type of anesthesia^c^			
General	27 (54.0%)	44 (60.3%)	
Regional	1 (2.0%)	0	0.408^b^
Combined	22 (44.0%)	29 (39.7%)	
Intraoperative anticholinergic load^c^	10 (62.5%)	22 (62.9%)	0.980^b^
Intraoperative clonidine administration^c^	6 (12.2%)	15 (22.4%)	0.161^b^
Intraoperative norepinephrine administration^c^	44 (89.8%)	60 (89.6%)	0.966^b^
Duration of anesthesia (min)	423 (288;503)	298 (199;394)	< 0.001^a^
Stay on ICU^c^	43 (82.7%)	38 (51.4%)	< 0.001^b^
Preoperative CRP mg/l	8.8 (3.6;28.2)	4.0 (1.4;14.7)	0.009^a^
Preoperative leukocyte counts/nl	6.4 (4.7;7.8)	5.5 (4.5;7.1)	0.334^a^
Postoperative CRP mg/l	72.5 (43.4;110.8)	49.7 (26.9;60.0)	0.026^a^
Postoperative leukocyte counts/nl	10.4 (8.3;13.1)	9.5 (7.6;12.8)	0.368^a^
Preoperative AChE activity U/gHb	44.3 (39.8; 48.2)	46.7 (42.5; 50.3)	0.060^a^
Preoperative BuChE activity 100 U/l	24.6 (18.8; 28.8)	25.6 (20.7; 30.6)	0.104^a^
Postoperative AChE activity U/gHb	42.3 (39.4; 48.6)	45 (41.9; 48.7)	0.056^a^
Postoperative BuChe activity 100 U/l	19.7 (14.8; 26.6)	22.5 (17.9; 26.4)	0.107^a^

Data are given as median with limits of the interquartile range (25th;75th percentile) or absolute frequency with percentage. A *p* value ≤ 0.05 was considered statistically significant; ^a^Mann-Whitney U test, ^b^chi-square test, ^c^missing data on frailty status: *n* = 2, on preoperative neurocognitive disorder: *n* = 23, on ADL: *n* = 2, on IADL: *n* = 14, on MNA: *n* = 2, on smoking behavior: *n* = 14, on alcohol consumption: *n* = 15, type of anesthesia: *n* = 4, intraoperative anticholinergic load: *n* = 76, clonidine/norepinephrine administration: *n* = 11, stay on ICU: *n* = 1, preoperative CRP: *n* = 1, preoperative leukocytes: *n* = 8, postoperative CRP: *n* = 92, postoperative leukocytes: *n* = 10

*MMSE* Mini-Mental-State-Examination, *BMI* Body-Mass-Index, *ASA-PS* American Society of Anesthesiologists’ physical status classification, *CCI* Charlson Comorbidity Index, *ADL* activities of daily living, *IADL* instrumental activities of daily living, *MNA* Mini-Nutritional Assessment, *GDS* Geriatric Depression Scale, *ICU* Intensive Care Unit, *CRP* C-Reactive Protein, *AChE* acetylcholinesterase, *BuChE* butyrylcholinesterase, *U* Units, *Hb* hemoglobin

**Fig. 2:**
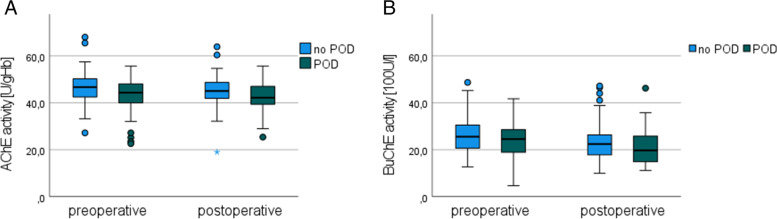
Pre- and postoperative cholinesterase activities in patients with (green) and without (blue) postoperative delirium. **A** AChE activity in U/gHb and **B** BuChE activity in100U/l. *AChE* acetylcholine esterase, BuChE butyrylcholine esterase, *POD* postoperative delirium, *U* Units, *Hb* hemoglobin

Patients with POD had lower preoperative AChE and BuChE activity compared to patients without POD but without statistical significance (see Fig. 2). Regarding postoperative cholinesterase activities, there were also no significant differences between the groups in terms of AchE and BuCHE activity. In addition, there was no association between AChE and BuCHE activities and subtypes of POD (see Additional file 1).

The relative change from pre- to postoperative activity (∆) did not differ between the groups regarding AChE or BuChE activity, but here was a high dispersion of the values. In the group without POD, AChE activity decreased by a median of 3.5% from pre- to postoperative (− 0.081; 0.057), while BuChE activity increased by a median of 12% (0.0001; 0.230). In the POD group, both enzyme activities decreased in median, AChE activity by 0.2% (− 0.110; 0.089), BuChE activity by 5.3% (− 0.053; 0.280).

The logistic regression analysis, adjusted for age, duration of surgery and CCI, showed that with 1 U/gHb higher preoperative AChE activity the odds of suffering POD relatively decreased by 5% (OR [CI] = 0.95 [0.89–1.00]. There was also a statistically significant association between postoperative AChE activity with the occurrence of POD after adjustment for confounders. At 1 U/gHb higher postoperative AChE activity the chance of developing POD decreased by 6% (0.94 [0.89–1.00], *p* = 0.05).

There was no statistically significant association between preoperative or postoperative BuChE activity with the occurrence of POD (Table 2). Due to external conditions, 19 of the 127 patients (15%) had their first postoperative blood sample taken on the third postoperative day. For postoperative BuChe activity, the distribution of values was the same in both groups; it did not make a statistically significant difference whether the measurement was performed on the first or the third postoperative day. Concerning postoperative AChE activity, it made a significant difference whether blood collection happened on the first or third postoperative day. Therefore, the logistic regression analysis was repeated with this subgroup of patients *n* = 108 (see Table 2). Again, the postoperative AChE activity was associated with the occurrence of POD. The OR was 0.93 (0.87–1.00) with a slightly wider confidence interval. There was no clear association between postoperative BuChE activity and the occurrence of POD.

**Table 2:** Association of postoperative AChE / BuChE activity with POD: *n* = 127 versus *n* = 108

	OR (CI 95%)	*p*-value	R^2^
AChE activity postop. (U/gHb) (*n* = 108)	**0.930 (0.867–0.998)**	**0.044**	**0.216**
AChE activity postop. (U/gHb) (*n* = 127)	0.943 (0.890–1.000)	0.050	0.217
BuChE activity postop. (100 U/l) (*n* = 108)	**0.988 (0.936–1.043)**	**0.663**	**0.172**
BuChE activity postop. (100 U/l) (*n* = 127)	0.985 (0.937–1.036)	0.56	0.185

*n* = 108 patients (blood assessment exclusively on first postoperative day)

*n* = 127 patients (blood assessment in 85% on postoperative day 1 and in 15% on third postoperative day)

Dependent variable: POD, independent variables: postoperative AChE- or BuChE- activity, age, duration of surgery, CCI. *OR* odds ratio, *CI* confidence interval, *R2* Nagelkerke’s pseudo measures, *AChE* acetylcholinesterase, *BuChE* butyrylcholinesterase, *U* Units, *Hb* hemoglobin, *POD* postoperative delirium, *CCI* Charlson Comorbidity Index

## Discussion

In summary, we found an association between pre- and postoperative AChE activity and the occurrence of POD. Pre- and postoperative BuChe activity was not associated with the occurrence of POD. We neither found an association between POD and the change of AChE / BuChE activity from pre- to postoperative. Furthermore, it was found that the timing of the postoperative blood collection had an influence on the AChE activity.

Overall, 41% suffered from POD. Generally speaking, the incidence of POD varies in different studies within a broad range [29]. Compared to others, POD rates in our study population were pretty high [29]. For example, in another study with 190 patients undergoing major non cardiac surgery, only 15.3% of the patients suffered POD [30]. The average age in this population was even higher compared to our study group. It is difficult to compare preoperative risk factors such as comorbidities when they are described with different scores. One main difference between the study populations was duration of anesthesia. It is reasonable, that intraoperative risk factors, e.g. bleeding, influence of anticholinergic medication etc. are associated with longer duration of surgery and anaesthesia [6]. Since our patients had longer duration of anesthesia (423 min.) compared to the previously mentioned study group (303 min.), this might be one important factor explaining the different delirium rates. Patients with POD suffered significantly more often from preoperative frailty. They also underwent malignant tumor surgery more often than patients without POD. From our point of view, these two aspects are interrelated. Patients with malignant diseases are more likely to be frail, since cancer diseases often lead to loss of weight, fatigue, anemia and general weakness. This fact might also explain, why the group of POD patients was more often treated on the ICU. Not only was their general health condition preoperatively worse, they also underwent longer and more complex abdominal tumor surgery.

In our study, 35 patients (71%) showed a hypoactive, 5 patients (10%) a hyperactive and 5 patients (10%) a mixed form of POD. Our findings are consistent with a prior evaluation of delirium subtypes which revealed that in surgical and trauma patients, hypoactive delirium (64 and 60%, respectively) was significantly more prevalent than the mixed (9 and 6%) and the pure hyperactive delirium (0 and 1%) [31]. A large systematic review and meta-analysis on incidence and prevalence of ICU delirium subtypes also showed that the majority of delirious ICU patients had hypoactive delirium [32]. The pooled prevalence of delirium subtypes were hyperactive (4% [95% CI, 3–6]), hypoactive (17% [95% CI, 13–22]), and mixed forms (10% [95% CI, 6–16]) [32]. Another systematic review revealed, that in studies with high average age (older than 65 years) patients were more likely to suffer from hypoactive delirium [33]. The high percentage of hypoactive delirium subtypes in our study might be explained due to surgical procedure and older patient age. Finally, we could not find an association between AChE and BuChE activities and subtypes of POD.

Previously published investigations about the association between cholinesterase activities and POD showed heterogeneous results. This can be explained by different inclusion criteria. In studies that included patients of at least 18 years, no difference in AChE activity was observed between patients with and without POD [15, 34, 35]. In cohorts including older adult patients and higher mean patient ages, an association between AChE activity and POD was observed more frequently [36–38]. There seems to be less differences in younger study populations regarding AChE activity between patients with and without POD. The cholinergic system of younger patients might be more resilient to external influences, such as anesthesia, surgery or drugs. Thus, enzyme activities might less likely to be affected. Already in 2010, the cholinergic deficiency hypothesis was discussed in a review [39]. Here, factors which are involved in cholinergic deficiency were summarized: impaired ACh synthesis, ischemia and global stressors as well as neurotransmitter imbalance [39]. These factors are primarily present in older adults patients. Furthermore, in animal experiments, it was observed that cholinergic neurons in the basal forebrain of older rats were more vulnerable to external, toxic influencing factors [40]. These cells, compared to neuron populations in other areas of the CNS, have a particularly high metabolism. With increasing age, the compensation might become insufficient [40]. This might explain why the studies showed different results depending on the average age of the study population. Whereas in younger, healthy patients, external stimuli are likely to evoke less severe changes in cholinergic metabolism, in older patients, due to age-related impaired cerebral metabolism, intraoperative central nervous deficits might happen more frequently. Nevertheless, POD also occurs in younger patient groups. It is a common complication in pre-school age children (age 5–7 years) and it can currently not be determined, whether this is due to age-related psychological issues or to additional inflammatory effects on the brain [6].

There is no evidence that cholinesterase activities are generally reduced when considering chronological age [41]. However, it has been observed that within the group of older adult patients, those with higher frailty had lower cholinesterase activity [41], which leads to the presumption that biological age may influence ChE activity. Consistent with the above considerations, the cholinergic system of older adult patients would already be impaired preoperatively, due to natural, age-related neurodegeneration, which varies interindividually and may result in cholinergic hypofunction [40]. This hypofunction may still be compensated preoperatively, but increases the patient’s vulnerability. An impairing event could exhaust the cholinergic system and lead to decompensation in older adult patients. This decompensation could result in decreased postoperative AChE activity and manifest itself clinically as POD. Such an event may be abdominal surgery, as in our study. Intra- and perioperatively, several factors affect the cholinergic system. During abdominal surgery, visceral parasympathetic fibers are manipulated. They are involved in immunologic mechanisms and communication between the periphery and CNS [8]. It is possible that the damage of parasympathetic nerve fibers during surgery disrupts direct neuronal transmission of information. Consequently, anti-inflammatory mechanisms regulated by these nerve fibers would also be affected. For this, information on surgical techniques (laparoscopic vs. open approach) would be essential. In line with this, patients suffering from POD had significantly higher pre- and postoperative CRP levels. These findings emphasize the important role of immunological mechanisms in the development of POD.

Prolonged surgical time has previously been identified as a risk factor for the occurrence of POD [6]. The duration of surgery was also independently associated with the occurrence of POD in our analysis. There is no precise data yet on the postoperative course of cholinesterase activities. Moreover, it is not clear when exactly they reach a minimum postoperatively. In our study, the time of postoperative measurement had a statistically significant impact on the distribution of postoperative AChE activity. Values were lower in the group of patients in which blood samples were taken on postoperative day 3. The groups were unequally distributed (108 vs. 19 patients) and both, standard deviation and confidence interval were larger in the smaller group.

In a trial published in 2017 with 217 cardiac surgical patients with an average age of 65 years, cholinesterase activities were measured on the first, second and third postoperative days [35]. AChE activity was lowest on the first postoperative day in both patients with and without POD. In contrast, BuChE activity was lowest on the third postoperative day in both groups. Adam et al. performed cholinesterase activity measurements preoperatively and on the first and second postoperative days after elective cardiac surgery in 114 patients with an average age of 69 years [42]. They observed that the medians of both enzyme activities were lowest on the first postoperative day in both patients with and without POD. These observations indicate that course dynamics of cholinesterase activities may be very heterogeneous. The factors influencing the dynamics of cholinesterase activities are poorly understood. In animal experiments, the neuronal release of ACh was subject to a circadian rhythm [43–45]. Thus, it may not only be relevant on which postoperative day, but also on what time of day. It is challenging to determine the best time for measurement of cholinesterase activities.

Taken together, in future investigations time-depending fluctuations, interrelations between circadian rhythm and enzyme activities as well as information on surgical techniques and perioperative drug administration should be considered.

### Strength and limitations

A key strength of this investigation is the prospective, precise, standardized and validated assessment of POD. The study data base provided extensive information on possible confounders. However, high rates of missing values for preoperative neurocognitive disorder, postoperative CRP levels, intraoperative anticholinergic load, smoking behavior and alcohol consumption might have had an effect on the results. Also, some other important limitations should be considered. In 40 of the 167 patients undergoing abdominal surgery, pre- and postoperative cholinesterase activity measurements were not completely assessed. A larger study population would have provided further insights, as well as additional data concerning surgical techniques (laparoscopic vs. open approach) or amount of intraoperative opioids or hypnotics.

Furthermore, the preoperative blood sampling was performed in the morning of the surgery. At this point, there might already have been influences on the cholinesterase activities, for example by taking premedication, fasting, stress and anxiety. Since blood samples were taken only once per day, the circadian rhythm of the enzyme activities could not be taken into account. There was no exact documentation after how many minutes after collecting the blood sample the testing was performed. In addition, repetitive pre- and postoperative measurements would have reflected the intra- and interindividual courses in a better way.

## Conclusions

Our analysis found an association between pre- and postoperative AChE activity and the development of POD in older adult patients undergoing abdominal surgery. Our approach was exploratory and hypothesis-generating. Our results support the hypothesis that the cholinergic system might play a role in the pathophysiology of POD in abdominal surgical patients. It remains unclear, whether AChE activity might be considered as a potential marker for neuroinflammatory processes. However, in addition to circadian rhythmicity and anticholinergic drugs, there are probably other, possibly unknown, influencing factors on AChE activity. Manipulation of visceral nerve fibers has been discussed as one of them.

## Supplementary Information

**Additional file 1: Additional Fig.** **1.** Preoperative AChE activity regarding POD subtypes. **Additional Fig.** **2.** Postoperative AChE activity regarding POD subtypes. **Additional Fig. 3.** Preoperative BuChE activity regarding POD subtypes. **Additional Fig. 4.** Postoperative BuChE activity regarding POD subtypes.

## Data Availability

The datasets generated and analyzed during the current study are not publicly available as no consent for this was obtained from participants but are available from the corresponding author on reasonable request.

## Abbreviations

ACh: acetylcholin
AChE: acetylcholine esterase
ADL: activities of daily living
ADS: Anticholinergic Druc Scale
ASA-PS: American Society of Anesthesiologists’ physical status classification system
BioCog: Biomarker Development for Postoperative Cognitive Impairment in the Elderly
BMI: Body Mass Index
BuChE: butyrylcholine esterase
CAM: Confusion Assessment Method
CAM-ICU: Confusion Assessment Method - Intensive Care Unit
CCI: Charlson Comorbidity Index
CCM: Campus Charité Mitte
CRP: C-Reactive Protein
CVK: Campus Virchow Klinikum
DSM-5: American Psychiatric Association’s fifth edition of the Diagnostic and Statistical Manual of Mental Disorders
GDS: geriatric depression scale
IADL: instrumental activities of daily living
ICU: Intensive Care Unit
MMSE: Mini-Mental-State-Examination
MNA: Mini-Nutritional Assessment
Nu-DESC: Nursing Delirium Screening Scale
OR: Odds Ratio
POD: postoperative delirium
postop.: postoperative(ly)
SD: standard deviation

## References

[1] Association, A.P., Diagnostic and statistical manual of mental disorders (Dsm-5®), 5th ed. Washington, DC in Amer Psychiatric Pub Inc. 2013.

[2] Kat MG (2008). Long-term cognitive outcome of delirium in elderly hip surgery patients. A prospective matched controlled study over two and a half years. Dement Geriatr Cogn Disord.

[3] Bellelli G (2014). Duration of postoperative delirium is an independent predictor of 6-month mortality in older adults after hip fracture. J Am Geriatr Soc.

[4] Lundstrom M (2003). Dementia after delirium in patients with femoral neck fractures. J Am Geriatr Soc.

[5] Inouye SK, Charpentier PA (1996). Precipitating factors for delirium in hospitalized elderly persons. Predictive model and interrelationship with baseline vulnerability. JAMA.

[6] Aldecoa C (2017). European Society of Anaesthesiology evidence-based and consensus-based guideline on postoperative delirium. Eur J Anaesthesiol.

[7] Goehler LE (2000). Vagal immune-to-brain communication: a visceral chemosensory pathway. Auton Neurosci.

[8] Tracey KJ (2002). The inflammatory reflex. Nature.

[9] van Gool WA, van de Beek D, Eikelenboom P (2010). Systemic infection and delirium: when cytokines and acetylcholine collide. Lancet.

[10] Tune LE (1981). Association of postoperative delirium with raised serum levels of anticholinergic drugs. Lancet.

[11] Shytle RD (2004). Cholinergic modulation of microglial activation by alpha 7 nicotinic receptors. J Neurochem.

[12] Borovikova LV (2000). Vagus nerve stimulation attenuates the systemic inflammatory response to endotoxin. Nature.

[13] Maldonado JR (2013). Neuropathogenesis of delirium: review of current etiologic theories and common pathways. Am J Geriatr Psychiatry.

[14] Heinrich M (2021). Association between genetic variants of the cholinergic system and postoperative delirium and cognitive dysfunction in elderly patients. BMC Med Genet.

[15] Plaschke, K., et al. The Association of Blood Cholinergic Esterases and Other Risk Factors on the Development of Postoperative Delirium. 2017.

[16] Soreq H, Seidman S (2001). Acetylcholinesterase--new roles for an old actor. Nat Rev Neurosci.

[17] Taylor P, Radic Z (1994). The Cholinesterases: from genes to proteins. Annu Rev Pharmacol Toxicol.

[18] Reale M (2018). Butyrylcholinesterase and Acetylcholinesterase polymorphisms in multiple sclerosis patients: implication in peripheral inflammation. Sci Rep.

[19] Heinrich M (2021). Preoperative medication use and development of postoperative delirium and cognitive dysfunction. Clin Transl Sci.

[20] Charlson ME (1987). A new method of classifying prognostic comorbidity in longitudinal studies: development and validation. J Chronic Dis.

[21] Mahoney FI, Barthel DW (1965). Functional evaluation: the Barthel index. Md State Med J.

[22] Lawton MP, Brody EM (1969). Assessment of older people: self-maintaining and instrumental activities of daily living. Gerontologist.

[23] Guigoz Y, Vellas B, Garry PJ (1996). Assessing the nutritional status of the elderly: the Mini nutritional assessment as part of the geriatric evaluation. Nutr Rev.

[24] Saunders JB (1993). Development of the Alcohol Use Disorders Identification Test (AUDIT): WHO Collaborative Project on Early Detection of Persons with Harmful Alcohol Consumption--II. Addiction.

[25] Yesavage JA (1982). Development and validation of a geriatric depression screening scale: a preliminary report. J Psychiatr Res.

[26] Carnahan RM (2006). The anticholinergic drug scale as a measure of drug-related anticholinergic burden: associations with serum anticholinergic activity. J Clin Pharmacol.

[27] Worek F (1999). Improved determination of acetylcholinesterase activity in human whole blood. Clinica Chimica Acta Int J Clin Chem.

[28] Peterson JF (2006). Delirium and its motoric subtypes: a study of 614 critically ill patients. J Am Geriatr Soc.

[29] Androsova G (2015). Biomarkers of postoperative delirium and cognitive dysfunction. Front Aging Neurosci.

[30] Leung JM (2007). Apolipoprotein E e4 allele increases the risk of early postoperative delirium in older patients undergoing noncardiac surgery. Anesthesiology.

[31] Pandharipande PP (2007). Motoric subtypes of delirium in mechanically ventilated surgical and trauma intensive care unit patients. Intensive Care Med.

[32] Krewulak KD (2018). Incidence and prevalence of delirium subtypes in an adult ICU: a systematic review and Meta-analysis. Crit Care Med.

[33] la Cour KN (2022). Distribution of delirium motor subtypes in the intensive care unit: a systematic scoping review. Crit Care.

[34] Muller A (2019). Relevance of peripheral cholinesterase activity on postoperative delirium in adult surgical patients (CESARO): a prospective observational cohort study. Eur J Anaesthesiol.

[35] John M (2017). Acetylcholinesterase and butyrylcholinesterase in cardiosurgical patients with postoperative delirium. J Intensive Care.

[36] Cerejeira J (2011). Low preoperative plasma cholinesterase activity as a risk marker of postoperative delirium in elderly patients. Age Ageing.

[37] Zhao B, Ni Y, Tian X (2019). Low plasma cholinesterase activity is associated with postoperative delirium after noncardiac surgery in elderly patients: AProspective observational study. Psychosomatics.

[38] White S (2005). Enzymes of drug metabolism during delirium. Age Ageing.

[39] Hshieh TT (2008). Cholinergic deficiency hypothesis in delirium: a synthesis of current evidence. J Gerontol A Biol Sci Med Sci.

[40] Schliebs R, Arendt T (2011). The cholinergic system in aging and neuronal degeneration. Behav Brain Res.

[41] Hubbard RE (2008). Plasma esterases and inflammation in ageing and frailty. Eur J Clin Pharmacol.

[42] Adam EH (2020). Cholinesterase alterations in delirium after cardiosurgery: a German monocentric prospective study. BMJ Open.

[43] Davis B, Sadik K (2006). Circadian cholinergic rhythms: implications for cholinesterase inhibitor therapy. Dement Geriatr Cogn Disord.

[44] Jiménez-Capdeville ME, Dykes RW (1993). Daily changes in the release of acetylcholine from rat primary somatosensory cortex. Brain Res.

[45] Marrosu F (1995). Microdialysis measurement of cortical and hippocampal acetylcholine release during sleep-wake cycle in freely moving cats. Brain Res.

