# Supplementary material for: Association of cholinesterase activities and POD in older adult abdominal surgical patients
Source: BMC Anesthesiol. 2022 Sep 16;22:293. doi: 10.1186/s12871-022-01826-y (PMC9479414; doi:10.1186/s12871-022-01826-y)
Supplement: Supplementary file 1 — Additional file 1: Additional Fig. 1. Preoperative AChE activity regarding POD subtypes. Additional Fig. 2. Postoperative AChE activity regarding POD subtypes. Additional Fig. 3. Preoperative BuChE activity regarding POD subtypes. Additional Fig. 4. Postoperative BuChE activity regarding POD subtypes. [file 12871_2022_1826_MOESM1_ESM.docx]

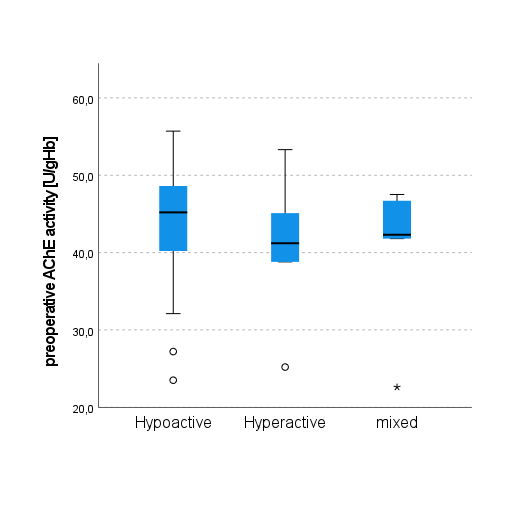


**Additional Figure 1: Preoperative AChE activity regarding POD subtypes.**
Differences were calculated using the Kruskal-Wallis-Test.

AChE – acetylcholine esterase, POD – postoperative delirium


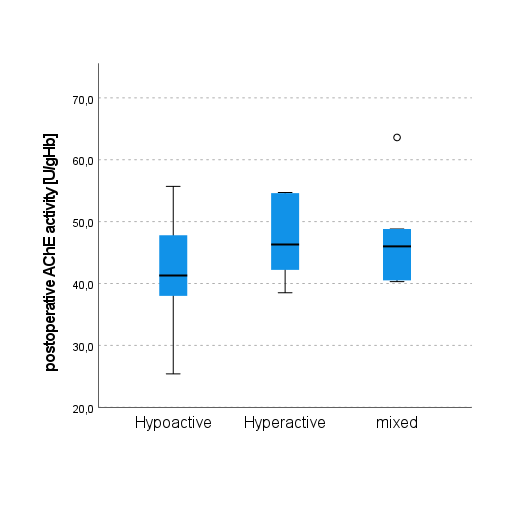


**Additional Figure 2: Postoperative AChE activity regarding POD subtypes.**
Differences were calculated using the Kruskal-Wallis-Test.

AChE – acetylcholine esterase, POD – postoperative delirium


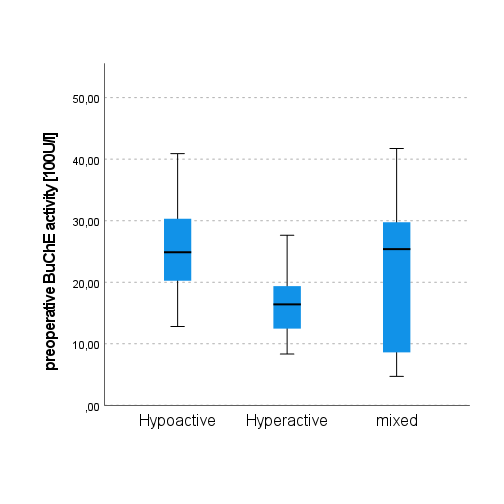


**Additional Figure 3: Preoperative BuChE activity regarding POD subtypes.**
Differences were calculated using the Kruskal-Wallis-Test.

BuChE – butyrylcholine esterase, POD – postoperative delirium


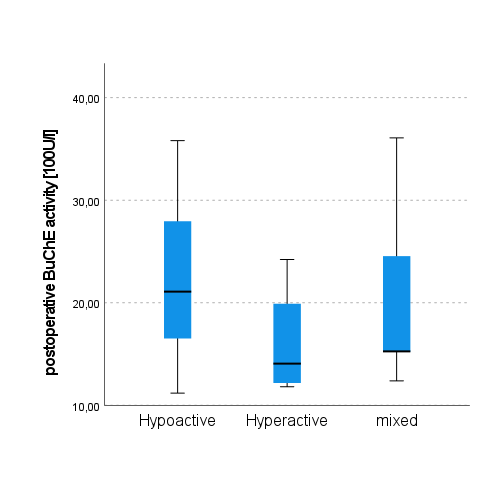


**Additional Figure 4: Postoperative BuChE activity regarding POD subtypes.**
Differences were calculated using the Kruskal-Wallis-Test.

BuChE – butyrylcholine esterase, POD – postoperative delirium
